# Bromide of Ethyl as an Anæsthetic*Read before Tri-State Medical Association of Alabama, Georgia, and Tennessee, October, 1891.

**Published:** 1892-02

**Authors:** E. H. Kuykendall

**Affiliations:** Chattanooga, Tenn.


### Southern Medical Record.

A MONHLY JOURNAL OF MEDICINE AND SURGERY.

VOL. XXII. ATLANTA, GA., FEBRUARY, 1892. No. 2

### Original Articles.

### *BROMIDE OF ETHYL, AS AN ANÆSTHETIC.

BY E. H. KUYKENDALL, M. D., CHATTANOOGA, TENN.

"The term anæsthetic, proposed by Dr. Oliver Wendell Holmes, means an agent capable of producing anæsthesia, or insensibility to pain."

It is true, anæsthesia is a term which, according to its etymological signification, should be applied to loss of sensation of touch, chiefly; and analgesia should be used to signify loss of sense of pain; however, the word anæsthesia, as expressive of the state of profound unconsciousness induced by anæsthetics, is now so firmly established by usage that it were better to retain it.

Insensibility to pain (analgesia) may be produced without simultaneous loss of common sensation, touch, as in the use of cocaine and carbolic acid.

Having administered Ethyl Bromide something over two-hundred and fifty times, and found it so extremely serviceable in the class of operations for which I make use of it, I bring this subject to the attention of this Association with the hope of doing something towards giving this valuable anæsthetic

* Read before Tri-State Medical Association of Alabama, Georgia, and Tennessee, October, 1891.

the place it should hold in our daily practice, alongside the other indispensable ones, chloroform and ether.

Ethyl Bromide, or Hydrobromic Ether, is a colorless liquid, volatile, having a fragrant odor and a hot, somewhat sweetish taste, afterward rather bitter. It is not inflammable. Its specific gravity is 1.420, and it boils at 104 Fahrenheit; readily decomposes on exposure to light and air, bromine being separated. It is freely soluble in alcohol and ether, but very sparingly in water.

As an anæsthetic, it was first known to Mr. Nunnely, of Leeds, and he first employed it in surgical practice in 1865. Dr. Turnbull gave an account of its properties, based on experimental and clinical evidence, in 1877. But the most extended trials of its anæsthetic powers were made by Dr. Levis, of Philadelphia, in 1879–80. In the latter year two unsuccessful cases occurred, one in the hands of Dr. Levis, its chief promoter, and the other in the practice of Dr. Marion Sims, of New York.

These fatal cases, and some crude physiological experiments undertaken to prove that ethyl bromide is a heart paralyzer, started a reaction against this anæsthetic, then beginning a promising career, and in a short time it fell very much out of use.

I have found it, however, to have valuable properties, which should preserve it from neglect, and bring it into more constant use.

In the fatal cases recorded, there are strong doubts in regard to the share of ethyl bromide in the results. In Dr. Levis' case, the patient was far advanced in pulmonary disease, and was unfit for the administration of any anæsthetic.

In Dr. Sims' case, the death of the patient occurred a number of hours after the operation, which was a long and tedious one, requiring very protracted use of the anæsthetic. To induce complete insensibility about zj must be administered rapidly. The odor is not unpleasant, and but little irritation of the air passages is produced.

If administered in full quantity, there is a very brief stage of excitement, hardly perceptible, and the stage of rigidity is very short and not pronounced. There is, practically, no irritation of the fauces.

The face is flushed, the ears are red, the eyes injected and watery from the increased lachrymation; the pupils more or less dilated. The action of the heart is accelerated and the pulse increases in force. The respiration is somewhat quickened. When the vapor of ether or chloroform is inhaled sense of faucial irritation and the need of air is experienced, also, more or less cough is produced.

The irritation of the fauces excites the flow of mucous, and the reflex act of swallowing. The first effect is a general exhileration; the pulse increases in frequency, the respiration becomes more rapid, and sometimes assumes a sobbing or convulsive character; the face flushes, talking, laughing, crying, singing, and sometimes praying indicate the cerebral intoxication.

This state of excitement varies in different individuals, and is more pronounced in character, and more persistent in the robust and rough natured male and the hysterical. At this period, although the patient can be easily aroused, sensibility to pain is decidedly diminished, although the sense of touch may be preserved, taste and smell are abolished and the sight is either abnormally acute, or is perverted by illusions.

If the inhalations be continued, the patient passes into the condition of complete insensibility. In women and children, and males reduced by illness, the production of insensibility is quietly attained, if the anæsthetics be not inhaled too rapidly. The stage of insensibility is preceded by a tetanic, convulsive stage, in which the voluntary muscular system and the respiratory muscles become rigid; the breathing stertorous, the face cyanosed.

This condition of rigidity is similar to, if not identical with, the tetanic stage of epileptic paroxysm.

If the inhalation be pushed still further, the tetanic rigidity subsides, the cynanosis disappears, the breathing proceeds quietly, and the state of complete muscular relaxation and loss of reflex movement is established.

At this time the surface is usually cool, and bathed with profuse perspiration, the countenance is placid, the eyes are closed, the pupils rather contracted than dilated, the respiration easy, but more shallow than normal; the pulse slower—it may be feebler, it may be stronger than in health, the functions of the cerebrum are suspended, only the centres presiding over respiration and circulation continues in áction.

The main deviations observed from these successive stages, when administering bromide of ethyl, are, that we have, practically, no stage of faucial irritation beyond the choky sensation; a scarcely noticeable stage of excitement, and the period of rigidity is very short and not pronounced ; no cerebral intoxication, breathing in early stage decidedly quickened, the face never cyanosed, but constantly flushed, the ears red, the eyes injected, with the pupils more or less dilated.

My mode of administration is to take a towel and form of it a closely fitting cone, having previously placed a layer of firm close paper (usually a leaf from a good quality printed pamphlet) between the folds.

I prefer a towel to all patent inhalers, for I have found none to so well exclude the admixture of air. My attention is then given to instructing the patient in the part he is to perform, to make it most comfortable to himself, and speedy in action. I wish to impress this point, for it is a most important one. I tell the patient it is going to feel choky, but that it will not choke him ; and the best way to get rid of the choky feeling, is just to blow it out with the mouth wide open. Now, of course, they cannot blow out, without taking the inspiration, but this seldom, if ever, occurs to the mind of the patient. I then practice the patient at blowing into the towel for half a minute before putting any anæsthetic in it, leaving this impression, that he must blow it out, the last one upon his mind. I then turn dr.j. (one drachm) of the ethyl bromide into the apex of the cone, and place it closely over the mouth and nose, telling them at the same time to blow it out. Usually three or four whiffs serve to produce loss of consciousness, and in a half minute (a minute at the longest) complete anæsthesia is produced. Just so soon as this is attained, I remove the towel altogether. The stage of complete anæsthesia lasts from a minute and a half to two minutes, in which time many painful operations can be completed.

The patient comes from under the influence of the anæsthetic as if waking from a natural sleep, with the mind perfectly clear and with complete contol of the muscular system.

If my instructions fail to have children inhale the bromide as I wish, and they hold their breath instead of breathing freely, I give the anæsthetic free from admixture of air for about half a minute and then remove the towel a little, to allow them to take one free inspiration of air, but as soon as this is done, I again place the towel over the mouth and nose, when the breathing always proceeds as desired, no further holding of the breath ever having been noted in my experience.

I have often noticed complete insensibility to pain to last for a minute or two after the patient has returned to consciousness, and diminished sensibility to keep up much longer.

Dr. Julian J. Chisholm, of Baltimore, with whom I have had my training in the use of this drug, has administered it some three thousand (3,000) times, and he, (in his larger experience,) as well as myself, has never seen the slightest indication of harmful results, following or accompanying this careful method of administration.

It is not to be used in long operations, but is eminently suitable for any that can be performed successfully in two minutes

I have found it sufficiently lasting to perform squint operations on very young children; to open into a mastoid abscess, perform optico-ciliary neurotomy, or enucleation of the eye-ball (having first taken up the muscles under cocaine,) and many minor to these.

If the operation is not completed when consciousness returns, after the first administration, or if seen that it will not be completed, do not repeat the administration of bromide, but keep up the anæsthesia with chloroform. When the bromide is repeated or the anæsthesia kept up with chloroform, nausea is certain to follow; whereas, it rarely follows the single administration of the ethyl bromide.

I have never given ether in conjunction with it, but always used chloroform, when a single administration of the ethyl bromide did not produce anæsthesia of sufficiently long duration in which to complete the operation.

Some claim it must be much more dangerous than chloroform or ether, from its power and rapidity of action, but they lose sight of the fact that nitrous oxide has also this rapid action, yet is universally conceded the softest of anæsthetics.

When giving an anæsthetic, it is well for the administrator to keep a sharp notice of the ears, for these are always flushed, but quickly pale on the slightest disturbance to circulation, affording one of the earliest warnings of danger. I have never found it necessary in my experience in giving anæsthetics, to use instruments, with their oftimes consequent laceration, for the purpose of getting the tongue out of the way, to secure free inspiration and expiration.

By forcible extention of the head, with the hand under the chin, by the action of the digastric, stylo-hyoid, mylo-hyoid, and genio-hyoid muscles in this position, the hyoid bone is lifted up, bringing of necessity the larynx with it, and gaining by this means the same end as pulling on the tongue itself.

